# Risk factors for enterobacter cloacae colonisation at a neonatal intensive care unit in the Netherlands

**DOI:** 10.1186/2047-2994-4-S1-P237

**Published:** 2015-06-16

**Authors:** J Hopman, I Maat, ED Jong, D Liem, WD Boode, A Voss, A Tostmann

**Affiliations:** 1 Medical Microbiology / Hygiene Infection Prevention, Radboud University Medical Centre, Nijmegen, Netherlands; 2 Medical Microbiology / Hygiene Infection Prevention, Radboud University Medical Centre, Netherlands; 3 Neonatology, Dept of Paediatrics, Radboud University Medical Centre, Nijmegen, Netherlands

## Introduction

Following an outbreak of *Enterobacter cloacae* complex ESBL at a neonatal intensive care unit (NICU) in a large tertiary care hospital in the Netherlands, a routine *E. cloacae* complex screening of all neonates was introduced. Literature on colonisation rates and risk factors for neonatal colonisation with *E. cloacae* are limited.

## Objectives

To determine the colonisation and risk factors for colonisation with *E cloacae* complex at the NICU.

## Methods

Neonates who were admitted at the NICU between March 2013 and April 2014 and who were screened for *E. cloacae* were included in this study. Microbiological screening data was extracted from the medical microbiology database. Demographical, clinical and admission data and information on birth weight and gestational age were extracted from the electronic patient records. Colonisation rates and risk factors were determined for all neonates and a subgroup of those with gestational age of 30 weeks or below.

## Results

Out of 353 neonates who were included in the study, 42 were positive for *E. cloacae* (11.9%; 95% confidence interval 8.8-15.6%). Risk factors for colonisation were: a lower birth weight, a lower gestational age, lower 1 minute APGAR score and longer duration of admission at the NICU. In neonates who were born ≤30 weeks of gestational age, longer duration of admission and lower gestational age remained independent risk factors for colonisation with *E cloacae*.

## Conclusion

The colonisation rates were highest in the most vulnerable neonates, i.e. those with a lower gestational age, lower birth weight and a longer admission at the NICU. These factors are all interrelated and part of (extreme) prematurity. In situations where screening is performed in response to a suspected outbreak, it is helpful to know the background colonisation rate in order to interpret the colonisation rates. When limited resources are available a cost effective strategy could be to limit screening to neonates born after pregnancy duration <30 weeks.

## Disclosure of interest

None declared.

